# Does Diet Have a Role in the Treatment of Alzheimer's Disease?

**DOI:** 10.3389/fnagi.2020.617071

**Published:** 2020-12-23

**Authors:** Mitchell Thelen, Holly M. Brown-Borg

**Affiliations:** Department of Biomedical Sciences, University of North Dakota School of Medicine and Health Sciences, Grand Forks, ND, United States

**Keywords:** aging, diet, interventions, Alzheimer's disease, lifespan

## Abstract

The aging process causes many changes to the brain and is a major risk factor for the development of neurodegenerative diseases such as Alzheimer's Disease (AD). Despite an already vast amount of research on AD, a greater understanding of the disease's pathology and therapeutic options are desperately needed. One important distinction that is also in need of further study is the ability to distinguish changes to the brain observed in early stages of AD vs. changes that occur with normal aging. Current FDA-approved therapeutic options for AD patients have proven to be ineffective and indicate the need for alternative therapies. Aging interventions including alterations in diet (such as caloric restriction, fasting, or methionine restriction) have been shown to be effective in mediating increased health and lifespan in mice and other model organisms. Because aging is the greatest risk factor for the development of neurodegenerative diseases, certain dietary interventions should be explored as they have the potential to act as a future treatment option for AD patients.

## Introduction

Aging is the major risk factor for many diseases but the factors that drive age-related changes and promote dysfunction are poorly understood. Slowing or even delaying these processes may lead to extended periods of health in humans. With each passing year, aging has a greater impact on the U.S. healthcare system. As of the year 2020, the older adult population (65 years and older) is comprised of 56 million people and is expected to increase to nearly 90 million by the year 2050 (Alzheimer's Association, 2020a). In fact, projections point toward the year 2030 as being the first time in U.S. history where the population of those over the age of 65 will outnumber those 18 years and younger (Mendiola-Precoma et al., 2016). Aging is associated with an increased risk of disease and death; therefore, an aging population will place increased burden on our healthcare system and will have a greater financial impact on individuals throughout the country (Rose, 2009; Mendiola-Precoma et al., 2016).

Aging occurs in nearly all organisms and is represented by a physiological decline in function. Decades of work by many scientists led to the identification of several hallmarks of aging, many of which impact the brain as will be later presented. Various versions of the list exist but many agree about the general contributors. However, despite the identified hallmarks, the exact mechanisms underlying or driving biological aging remain inadequate to develop therapies to truly combat aging as a whole. This has led to alternative hypotheses suggesting that random cellular dysfunctions may be the greatest contributor to aging, potentially explaining why organisms of the same genotype (monozygotic twins) who are raised in a common environment have significantly different life spans (Kirkwood et al., 2005). An aging population warrants increased attention toward understanding the mechanisms that drive the aging process. This includes considering therapies to slow, delay or prevent aging. Past and current treatment options have focused on interventions specific for one disease process at a time (such as FDA drug approval for specific diseases). This has been one approach contributing to the increase in population health over a span of decades, as evidenced by an increase in U.S. life expectancy from 69.9 to 78.9 years from the years 1959 to 2016. However, recent data suggests that U.S. life expectancy is reaching a plateau or even slightly decreasing (Woolf and Schoomaker, 2019). A greater understanding of aging offers vast potential toward the field of medicine. It provides hope for slower disease progression and increased life expectancy in humans. However, the ultimate goal is to lengthen the overall time individuals remain disease-free (increased health-span), which would have a major positive impact on individuals and the healthcare system.

## Brain Aging and AD

Aging impacts many organ systems, but the brain garners a large share of the interest and funding. The brain undergoes prominent structural and functional changes throughout a lifetime. Structurally, normal brain aging involves atrophy seen through reductions in both gray and white matter and an associated enlargement of the cerebral ventricles (Drayer, 1988). These age-related reductions in gray matter are most notable in the frontal and temporal lobes (Jack et al., 1997). Much of this normal brain atrophy appears to be due to neuronal loss, neuronal morphology changes, and dendritic and synaptic reductions (Terry and Katzman, 2001; Dumitriu et al., 2010). Other structural brain changes observed in normal aging include increased visualization of amyloid-β plaques, neurofibrillary tangles, white matter injuries, small-vessel ischemia, and microhemorrhages (Terry and Katzman, 2001; Park et al., 2013; Prins and Scheltens, 2015; Shim et al., 2015; Ramirez et al., 2016). Functional changes to the brain with increased age include a gradual decline in attention, memory, decision-making speed, learning, motor coordination, and sensory perceptions (Alexander et al., 2012; Dykiert et al., 2012; Levin et al., 2014). Cognitive function is most affected in the areas of executive function, working memory, and episodic memory (Alexander et al., 2012).

Aging is the greatest risk factor for developing many neurodegenerative diseases (Hou et al., 2019). Broad classification of neurodegenerative diseases includes amyloidoses, tauopathies, synucleinopathies, and TDP-43 proteinopathies (Dugger and Dickson, 2017). Alzheimer's Disease (AD), a mixed amyloidose and tauopathy pathology, is the most common neurodegenerative disease in the U.S. Currently, AD is five times more prevalent than Parkinson's Disease, the second most common neurodegenerative disease, and this margin is expected to continue increasing. To date, AD affects nearly five million Americans (1 in 10 people over the age of 65) and is the sixth leading cause of death in the U.S. These numbers are expected to grow as 13.8 million people are projected to be living with AD by the year 2050. Furthermore, AD and related dementias will cost the U.S. over $300 billion in 2020 with an expectation to increase to more than $1 trillion by the year 2050 (Alzheimer's Association, 2020a).

Despite great effort from the scientific community, the primary cause of AD remains unknown. Risk factors for developing familial AD (leading to early onset symptoms) include mutations in the amyloid precursor protein (APP), presenilin 1 and presenilin 2 genes (Bekris et al., 2010). However, risk factors for the much more common sporadic development of AD include increased age and mutations to the ApoE4 allele (Liu et al., 2013). Features of an AD brain involve noticeable amyloid-beta (Aβ) plaques and p-tau neurofibrillary tangles (Bloom, 2014). Aβ peptides are produced after the proteolysis of the type I integral membrane protein amyloid precursor protein (APP) (Esch et al., 1990; Shoji et al., 1992; Haass and Selkoe, 1993; Haass et al., 1993; Jarrett et al., 1993). Aβ plaque and p-tau neurofibrillary tangle formation starts with altered APP cleavage by β-secretases (BACE1) and γ-secretases to produce insoluble Aβ fibrils. These Aβ fibrils then polymerize into insoluble amyloid fibrils which aggregate into plaques (Fontana et al., 2004; Simunkova et al., 2019). Polymerization of Aβ fibrils cause activation of kinases which hyperphosphorylate microtubule associated p-tau and lead to their polymerization into insoluble neurofibrillary tangles. Accumulation of Aβ plaques and p-tau neurofibrillary tangles lead to microglial activation and are associated with neurotoxicity (Long and Holtzman, 2019).

Disruption of cerebral vasculature is associated with neuroinflammation in mice studies and has also been shown to contribute to aging and AD (Tarantini et al., 2017; Fulop et al., 2019). Regions of the brain with increased activity rely on a mechanism known as neurovascular coupling (NVC) to receive a compensatory uptick in regional oxygen and glucose. NVC works through the release of the vasodilating molecule nitric oxide (NO) from microvascular endothelial cells surrounding areas of metabolically active neurons and astrocytes (Toth et al., 2014, 2015; Tarantini et al., 2017, 2018, 2019a). Interruption of the NVC mechanism tends to be found in older adults and it also leads to cognitive impairment in mice (Fabiani et al., 2014; Tarantini et al., 2017, 2018, 2019a; Lipecz et al., 2019). In fact, in mouse models of aging, interventions which improve NVC and cerebral microvasculature also improve cognitive function (Csiszar et al., 2019; Tarantini et al., 2019b; Wiedenhoeft et al., 2019). Currently functional MRI (fMRI) is one method of measuring NVC in humans, yet many new technologies are being studied for their usefulness in monitoring the cerebral microvascular changes that accompany aging (Csipo et al., 2019; Lipecz et al., 2019).

AD is a slow progressing disease characterized by a decline in cognitive function. Recent estimates report the preclinical (asymptomatic) stage of AD occurs for up to 15 to 20 years prior to clinical symptom emergence (Morris et al., 2001; Sperling et al., 2011, 2013). This is based upon examinations of brains of older individuals, who die with no cognitive impairment or mild cognitive impairment, often revealing similar pathology to those with apparent AD (Mufson et al., 1999; Price and Morris, 1999; Bennett et al., 2005; Markesbery et al., 2006). AD is classified into early stage (mild), middle stage (moderate), and late stages (severe forms). Mild and moderate stages may last for years at a time as the individual's memory, cognitive ability, and ability to live independently slowly decline. By the late stage of AD, the individual loses their ability to respond to their environment, carry on a conversation, and eventually control movement. Memory and cognition also continue to decline during this time. Most people only live for 4 to 8 years following first diagnosis (Alzheimer's Association, 2020b).

A closer look at the specific hallmarks of aging as they relate to the central nervous system is warranted. The nine hallmarks of aging include genomic instability, telomere attrition, epigenetic alterations, loss of proteostasis, mitochondrial dysfunction, cellular senescence, dysregulated nutrient sensing, stem cell exhaustion, and altered intercellular communication and immune function (López-Otín et al., 2013). Many of these hallmarks are associated with neurodegenerative diseases such as AD. Studies have found increased DNA damage and altered DNA repair mechanisms in the brains of AD patients (Lovell et al., 1999). Certain epigenetic markers, such as methylation patterns in APP promoters of primates, are related to neurodegenerative features (Bradley-Whitman and Lovell, 2013). The mitophagy process seems to be defective in AD, such that stimulation of mitophagy in nematode and mouse models improved memory (Fang et al., 2019). Brain cells from AD models demonstrate reduced glucose and oxygen metabolic rates (Camandola and Mattson, 2017). Additionally, Aβ oligomers trigger senescence of neurons *in vitro* and senescent astrocytes, microglia, and neurons are found in greater numbers in human AD brains (He et al., 2013). *In vivo* and *in vitro* studies have found that APP expression on microglial cells is required for their proinflammatory activation to Aβ oligomers (Manocha et al., 2016). Thus, several aspects of the underlying pathology and clinical features found with AD, mirror those observed in the established hallmarks of aging.

Distinguishing normal changes to the brain with age vs. pathological changes remains a challenge to date. Difficulty arises from evidence indicating that many of the structural changes to the brain found in neurodegenerative diseases such as AD, resemble the structural changes observed in a normal aging brain. High levels of amyloid-β plaques, neurofibrillary tangles, synaptic loss, and neuroinflammation are hallmarks of AD. As previously discussed, these changes also comprise part of normal aging of the brain and it is not uncommon for these same features to be seen in a non-demented, older individual (Denver and McClean, 2018). However, many of the structural changes displayed in neurodegenerative diseases are more frequent and more severe than in the normal aging process (Park et al., 2013; Prins and Scheltens, 2015; Shim et al., 2015; Ramirez et al., 2016). Unfortunately, in the case of AD, by the time these structural changes have accumulated enough to begin manifesting in symptoms, the neuropathology is irreversible. A greater ability to distinguish AD pathology, especially in its early stages, vs. normal aging would lead to improved diagnostic capabilities and potential new drug discoveries (Denver and McClean, 2018).

## Therapies for AD

**Figure 1:**
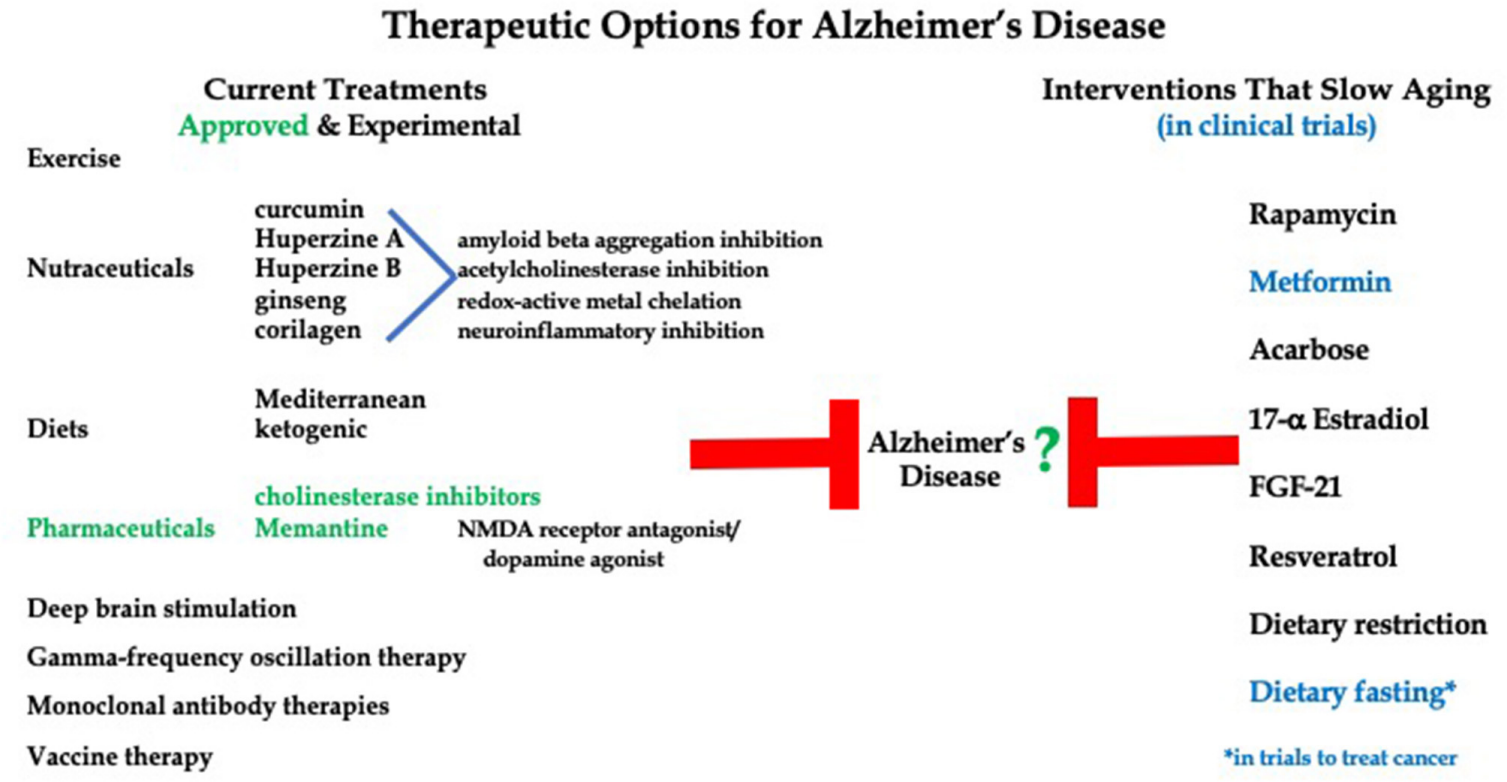
Current therapies to treat Alzheimer's Disease symptoms and pathologies versus potential therapies for AD that have been shown to delay aging.

Therapies to treat AD progression range from FDA-approved compounds to exercise, nutraceuticals and dietary interventions (Figure 1). Importantly, current treatment options for Alzheimer's Disease lack effectiveness. Only two therapeutic options are approved by the FDA, that being cholinesterase inhibitors (Donepezil, Rivastigmine, and Galantamine) and Memantine (an NMDA receptor antagonist/dopamine agonist) (Howard et al., 2012; Grossberg et al., 2013). Both options offer mild symptom management with no effect on long term progression of the disease. Additionally, over 20 compounds have reached large phase 3, double-blind, randomized control trials in cohorts of AD patients at various stages of disease progression, yet none have shown the ability to improve global functioning or slow cognitive decline (Long and Holtzman, 2019).

Due to the ineffectiveness of AD therapeutics, alternative treatment options are being explored. Huperzine A, a nutraceutical, has been shown to increase memory and activities of daily living in AD patients (Xing et al., 2014). This compound, along with other herbal drugs such as huperzine B, ginseng, corilagen, and curcumin offer therapeutic potential for AD due to their ability to cross the blood brain barrier (BBB) and act through the inhibition of acetylcholinesterase (AChE), chelation of redox-active metals, inhibition of the aggregation of Aβ and reduction of neuroinflammation (Simunkova et al., 2019). However, these drugs are not approved by the FDA and may suffer from lack of purity and potency (Xing et al., 2014). Recently, several small clinical studies have shown a relationship between a ketogenic diet and improved cognition in AD and other neurodegenerative disease patients (Rusek et al., 2019). Administration of medium-chain triglyceride-based ketogenic formula for 12 weeks to 20 patients with mild-to-moderate AD led to a significant increase in working memory, short-term memory, and processing speed. Specifically, these patients showed improvements in the digit-symbol coding test as well as the immediate and delayed logical memory tests (Ota et al., 2019). Many treatments for AD are designed to target the production and/or accumulation of Aβ and tau proteins, but this approach has not been promising so far. Monoclonal antibodies designed to target and remove abnormal amyloid beta have shown no improvement in cognitive function in early and late stage AD patients (Doody et al., 2014; Salloway et al., 2014; Honig et al., 2018). Potential vaccines against tau protein are currently under investigation as well as deep brain stimulation and gamma-frequency oscillation therapies (Weller and Budson, 2018; Koseoglu, 2019). Thus, although numerous therapies have been explored, very few clinical options are currently available to stop or slow progression of neurodegenerative disorders. There are numerous reports showing an association between diet, exercise, and reduced risk of developing neurodegenerative diseases, but these are not considered in this discussion of treatments for ongoing disease.

## Models of AD

AD is modeled in the laboratory to address the biology of specific cell types as well as in mice primarily, to study disease onset, progression, and treatment. Mouse models, including transgenic and non-transgenic options, are most common for the investigation of AD and offer a variety of pros and cons (Puzzo et al., 2015). Several transgenic mouse lines have been created to mimic human AD pathology. These animals are genetically engineered to overexpress one or more of the proteins found in human AD patients and often include APP, tau, and/or presenilin. One commonly used mouse line is the double transgenic APP/PS1 which develop a robust deposition of amyloid-beta plaques due to mutations in APP on chromosome 21 and presenilin 1 (PS1) on chromosome 14. These mice show increased levels of soluble Aβ40 and Aβ42 at an early age which allows for study of an AD-related phenotype (Puzzo et al., 2015).

Behavior studies of transgenic mice aim to assess the cognitive domains of these AD models in ways that are similar to what is observed in human AD. Alterations to working memory, executive function, and attention are common aspects of cognition which are disrupted in human AD and can also be studied in transgenic mice. In humans, working memory is assessed through verbal tasks, yet working memory is typically studied in mice through maze-type tasks in which spatial working memory is evaluated. AD transgenic mice show deficits in working memory by taking longer to learn where the food reward is located and which aspects of the maze they have previously visited (Webster et al., 2014). Executive function requires higher order cognition such as planning, reasoning, and cognitive flexibility and is observed in humans through tests such as the Wisconsin Card Sorting Task (Drewe, 1974; Robinson et al., 1980; Arnett et al., 1994). AD transgenic mice show deficits in executive function abilities through tasks involving set-shifting, reversal learning, and response inhibition (Zhuo et al., 2007; Papadopoulos et al., 2013; Romberg et al., 2013). The most widely used method of examining disruptions in attention for transgenic mice is through tasks, such as the five-choice serial-reaction time task (5-CSRTT), that utilize sustained attention divided among multiple spatial locations, where a large number of trials and errors of commission, omission, and reaction time are scored (Webster et al., 2014). These rodent tests most closely resemble Leonard's 5-CSRTT and Continuous Performance Tests (CPT) of sustained attention used in humans, however, there remains difficulty translating attention results from mice AD models to those seen in humans (Rosvold et al., 1956; Wilkinson, 1963; Young et al., 2009).

The study of AD through transgenic mice (including APP/PS1 mice) is limited in some ways. First, transgenic mice express a genetic form of AD while most cases in humans are not genetically based, but rather develop sporadically at older ages. Additionally, transgenic mutations introduced into mice are not able to match the complexity seen in AD such that no mouse models can reproduce the full spectrum of human AD symptoms and pathology (Tai et al., 2017). Non-transgenic models of AD typically involve molecular (such as Aβ or tau) intracerebroventricular or intrahippocampal injections into mice. This method of investigation offers the benefit of studying acute effects on brain tissue of Aβ or tau exposure, which is more like the sporadic development of AD pathology typically occurring in humans when compared to transgenic mice. Yet, non-transgenic mice do not reproduce the gradual rise of Aβ that occurs over many years in humans and it does not replicate the spreading of pathology through regions of the brain, which is a major component of AD pathology (Puzzo et al., 2015). Ultimately, while there remains uncertainty as to how similar pathogenic pathways found in mice are to those in humans, mouse models are a valuable tool for the study of the AD process (Shineman et al., 2011).

## Therapies for Aging

Currently, there are no FDA approved interventions to slow and/or delay aging. However, the search is on for drugs that may alter the aging process in humans. To date, several drugs presently approved and prescribed to treat symptoms or slow specific disease processes have shown the ability to also slow aging in model systems. Examples include Rapamycin, Metformin and Acarbose. The advantage of studying these medications is that their safety for use in humans is already known. Notable non-FDA approved options also under current study, include 17α -Estradiol (17α-E2), Fibroblast-growth factor-21 (FGF21), and Resveratrol. Further, interventions targeting the sirtuins, NAD biosynthesis, amino acids, autophagy, and senescence pathways have shown promise as well and are actively being investigated in models of aging and health (Gonzalez-Freire et al., 2020).

Rapamycin use is FDA approved for its immunosuppressive and anti-graft rejection properties (Camardo, 2003). However, Rapamycin and other rapalogs have shown an ability to increase lifespan in animal studies through inhibition of the mammalian target of rapamycin (mTOR) pathway. The mTOR pathway is a metabolic regulator whose activation is triggered by the insulin/IGF-1 axis, amino acid levels, and glucose levels which all signal cellular energy status. Signals act through two multiprotein complexes (mTORC1 and mTORC2) and lead to different outcomes. In short, mTORC1 activation leads to protein translation and cell growth, whereas its inhibition blocks growth and induces stress response pathways, such as autophagy, leading to pro-longevity effects (Laplante and Sabatini, 2012; Saxton and Sabatini, 2017). Pharmacological mTORC1 inhibition extends lifespan in mice and involves multiple processes including autophagy, lipid synthesis, mitochondrial metabolism, ribosomal biogenesis, and modulation of the senescence-associated secretory phenotypes (Pan and Finkel, 2017). Contrarily, mTORC2 inactivation is believed to be responsible for the unwanted insulin resistance associated with rapamycin treatment (Saxton and Sabatini, 2017). Inhibition of mTORC1 occurs following both acute and chronic administration of rapamycin while the unwanted inhibition of mTORC2 requires long-term rapamycin treatment (Li J. et al., 2014). Thus, rapamycin and other rapalogs remain potential candidates for use to interfere with aging processes and extend health span.

Metformin is another drug currently approved for use in humans that has shown promising age-altering effects in animal studies. Metformin is a safe biguanide-class drug used as the first line defense for type II diabetes in humans. Its effectiveness in type II diabetes management comes from its ability to lower hepatic glucose production and insulin resistance. However, there are reports that metformin use seems to offer other health benefits, one of which is its anti-aging properties. Administration of metformin leads to caloric restriction-like benefits such as improved insulin sensitivity, AMP-activated protein kinase (AMPK) activity, and better antioxidant protection (Martin-Montalvo et al., 2013). Neonatal mice injected with a single dose of metformin on the 3rd, 5th, and 7th days after birth showed a significant increase in lifespan for males and a slight (non-significant) increase in females (Anisimov et al., 2015). In both male and female neonatally-treated mice, hormonal and metabolic serum testing was unaltered compared to control. However, metformin-treated males had decreased body weight plus food and water consumption compared to control mice. Female treated mice showed no such difference compared to controls (Anisimov et al., 2015). Contrarily, administration of metformin to 2-year-old male mice leads to improved health without increased lifespan (Alfaras et al., 2017). Based on its promising results from cellular and animal studies on aging and its known safety in humans, a ground-breaking clinical trial termed TAME (Targeting Aging with Metformin) is underway. TAME is the first drug trial approved by the FDA that directly targets aging. The study plans to examine metformin's ability to delay age-related diseases beyond its effects on glucose metabolism through the study of 3,000 subjects across the U.S. between the ages of 65 to 79. Results of TAME could have a profound impact on the health care and research community. If metformin shows the ability to modulate aging and related diseases, outside of its impact on diabetes, it would be the first step in the development of drugs specifically targeting the biology of aging (Barzilai et al., 2016).

Acarbose is an alpha-glucoside inhibitor used to treat hyperglycemia and type II diabetes. When consumed with a carbohydrate-rich meal, acarbose competitively inhibits complex carbohydrate breakdown along the brush border of the small intestine resulting in delayed dietary carbohydrate breakdown and absorption (Caspary and Graf, 1979). Administration of acarbose seems to have a positive impact on both short-term and long-term blood glucose and insulin levels while also receiving reports of decreased body weight in both animal and human studies (Brewer et al., 2016). In animal studies, acarbose administration leads to extended lifespan and improved health-span particularly in male mice through an increase in fibroblast growth factor-21 (FGF21) and a decrease in IGF-1 levels (Harrison et al., 2014).

17α -Estradiol (17α-E2), Fibroblast-growth factor-21 (FGF21), and Resveratrol lack FDA approved use in humans but studies of each have shown promise for an ability to curb detrimental effects of aging in mice. 17α-E2 is a non-feminizing hormone found in humans whose administration in mice leads to reduced body weight, extended lifespan, and mitigated metabolic and age-related chronic inflammation. However, these effects were limited to only male mice (Stout et al., 2017). Fibroblast-growth factor-21 (FGF21) is a protein hormone which attenuates GH/IGF1 signaling in mice (Mendelsohn and Larrick, 2012). Transgenic overexpression of FGF21 leads to increased lifespan (Zhang et al., 2012). Finally, Resveratrol is a polyphenol found in mulberries, peanuts, and red grapes. Supplementation of this polyphenol in monkeys has shown increased health while supplementation in C. Elegans leads to increased lifespan. There have been mixed results in human studies (Gonzalez-Freire et al., 2020). Currently, there are new compounds being tested and older drugs being repurposed for potential utilization to slow aging processes and extend health in older persons, but more studies are needed.

## Diet, Alzheimer's Disease and Aging

Since aging is the major risk factor for neurodegenerative disease, one therapeutic tactic could be to investigate agents that slow or delay aging processes and evaluate the impact on progression of AD and related dementias (Figure 1). Little research has looked at the potential role of diet on AD progression. There is evidence for certain dietary practices, such as Mediterranean diet (MD) and vitamin supplementation, offering protection against neurodegenerative disease development, but the effects of dietary intervention on the management of AD is relatively unknown (Mendiola-Precoma et al., 2016; McGrattan et al., 2019). A few small clinical studies have showed a relationship between ketogenic diet and improved cognition in AD patients (Rusek et al., 2019). These findings seem to be supported by studies showing AD patients consistently exhibit reductions in cerebral glucose utilization without alteration in brain ketone metabolism (Castellano et al., 2015; Taylor et al., 2019).

More specifically, diet has been proposed to directly affect many of the underlying features of AD progression–including amyloidogenesis, oxidative stress, and inflammation (Wu et al., 2004; Gómez-Pinilla, 2008; Murphy et al., 2014). Specific components of diet have been heavily studied for their potential role in the development and/or management of AD. For example, elevated levels of cholesterol cause increased amyloid beta production through the increased activity of APP cleaving enzymes γ-secretase and BACE1 as well as facilitating a conformational change from a helical-rich Aβ structure to an aggregation prone β-pleated sheet (Kakio et al., 2001; Thirumangalakudi et al., 2008; Xiong et al., 2008). Therapeutic lowering of cholesterol via statin therapies has also shown an association with decreased amyloid beta accumulation and AD development (Shepardson et al., 2011; Lin et al., 2015). However, a gradual decline in serum cholesterol is typically seen with dementias which shows the complexity of AD pathogenesis (Mielke et al., 2010; Presečki et al., 2011). Studies on fatty acids have yielded contradicting results, but generally saturated fats, trans-fats, and ω-6 fatty acids offer no benefit or may be detrimental in the context of AD while ω-3 fatty acids have demonstrated some potential benefits (Liyanage et al., 2019).

The association between a diet rich in carbohydrates and AD progression is strong and there is speculation as to whether altered glucose metabolism may be causative in AD. Consistently high levels of dietary sugars is linked with insulin resistance, which has been proposed as a contributive factor for AD development (Liyanage et al., 2019). Interestingly, brains of AD patients have shown decreased levels of the glucose transporters GLUT1 and GLUT3, which allow glucose to cross the blood brain barrier and provide energy to the CNS (Szablewski, 2017). Decreased glucose delivery to the brain leads to glucose starvation and stimulates AD neuropathologies, vascular degeneration, and impaired cognition (Iadecola, 2015; Winkler et al., 2015). Additionally, proper insulin signaling, which is disturbed in the AD brain, has also been suggested for appropriate regulation of amyloid beta and tau proteins (Hong and Lee, 1997; Qiu et al., 1998; Vekrellis et al., 2000; Planel et al., 2007; El Khoury et al., 2014). Insulin resistance also triggers inflammation, which may worsen AD pathology (Creegan et al., 2015).

In general, alterations to dietary protein intake modifies lifespan. Mice fed one-of-many diets varying in the ratio of macronutrients and total energy content (as seen in the Geometric Framework) found that those on low protein/high carbohydrate (LPHC) diets had the greatest improvements in longevity and metabolic health compared to all other diets (Solon-Biet et al., 2014; Le Couteur et al., 2016). In humans, a national representative study of nutrition involving a United States population (6,381 individuals aged 50 years and over) found that individuals who got more of their dietary calories from protein had significantly increased risk of all-cause cancer and cancer-related mortality. Interestingly, this relationship only applied to those under 66 years of age, as those older showed reduction in all-cause mortality when getting more of their calories from protein. The protective effect of increased dietary protein found in the older group may be explained by preventing sarcopenia and frailty development, which are more commonly observed with increased age (Levine et al., 2014). The source of protein may also be important, as a large cohort study reported an association between higher animal protein consumption and increased risk of all-cause mortality, when compared to plant-based protein intake (Song et al., 2016). Whether protein intake impacts AD directly is an area that is underexplored.

Adherence to a Mediterranean diet (MD) has been linked with improved health outcomes. Recent research is finding that MD may also offer cognitive and/or neurological benefits (Baumgart et al., 2015). MD consists of a high intake in fruits, vegetables, fish, nuts, monounsaturated fats, whole grains, and legumes while limiting intake of red meat, saturated fats, dairy, and refined grains. Results of many clinical trials have been suggestive, but not definite, for MD having a protective effect on dementias and improved cognitive performance with aging (Gardener and Caunca, 2018). The mechanism through which MD may be neuroprotective is not fully understood. MD has been shown to decrease rates of stroke and other vascular diseases (such as obesity, hypertension, and diabetes). Vascular events are associated with the development of dementia and may mediate a connection between MD and neuroprotection. Further, the antioxidants and anti-inflammatory agents found in MD have been hypothesized to play a protective role. Mouse models given extra virgin olive oil (which is high in monounsaturated fats) had improved cognitive performance and reduced beta-amyloid and tau proteins (Qosa et al., 2015). Additionally, neuronal cells treated with oleocanthal (a polyphenol found in extra virgin olive oil) led to reduced amyloid-beta oligomer-mediated astrocyte inflammation and synaptic proteins (Batarseh et al., 2017).

## Dietary Restriction and Nutrient Signaling

A common pathway involved in many interventions aimed at slowing aging processes is the somatotropic axis. Genetic alterations to this axis have dramatic effects on an organism's health and life span that is well-conserved evolutionarily. For example, low levels of growth hormone (GH) signaling, found in both Ames dwarf and growth hormone receptor (GHR) knockout mice, can extend the life of mice by over 50% (Brown-Borg, 2016). In humans with GHR deficiency and subsequent decreased GH signaling as observed in Laron syndrome (LS), protection from diabetes and fatal neoplasms occurs (Guevara-Aguirre et al., 2011). Additionally, studies of centenarians have found that low plasma levels of insulin-like growth factor-1 (IGF-1) predict survival in these long-lived people (Milman et al., 2014). Currently there are several classes of compounds approved for use in patients with acromegaly which inhibit the GH/IGF-1 axis (Trainer et al., 2000; Giustina et al., 2014). While these therapeutics have not been studied for their effects on aging and health span in humans, their safety and the known effects of decreased GH/IGF-1 levels on the health span of mice shows a promising area of future research (Longo et al., 2015).

Many dietary interventions have been found to lower signaling through the GH/IGF axis and thus positively impact health and lifespan. Reduced mTOR signaling is also frequently observed in models of extended lifespan from dietary manipulation (Papadopoli et al., 2019). It has long been known that diet impacts health in humans, but more recent work has now found new connections between diet and aging. The three most common dietary interventions include caloric restriction (CR), fasting, and methionine restriction. Each have been shown to increase lifespan in mice and will be discussed in further depth.

Protocols for caloric restriction (CR; aka dietary restriction) in rodents generally call for reduced total caloric intake by 20–50% compared to ad libitum food administration (Vaughan et al., 2018). The following of these guidelines generally produces a significant increase in lifespan in mice. However, further studies have observed that mice of differing genetic backgrounds are affected by CR differently. 42 different recombinant inbred strains of mice subjected to CR led to extended lifespan in nine strains, significantly reduced lifespan in four strains, and no significant change to 29 strains (Liao et al., 2010; Rikke et al., 2010). These results show a need for continued study of the pathways relating diet to health and aging. Current understanding seems to indicate that CR's beneficial effects on aging work through the key nutrient and stress-responsive metabolic signaling pathways including IIS/FOXO, mTOR, AMPK, sirtuins, NRF2, and autophagy (Hwangbo et al., 2020). Application of CR to human studies has begun. To an extent, chronic CR has shown to be beneficial to human health such that moderate CR without malnutrition causes a protective effect against obesity, type II diabetes, inflammation, hypertension and cardiovascular disease, all of which are major causes of morbidity, disability and mortality (Fontana et al., 2004). However, there remains valid concerns against CR since the duration and severity required for optimal health and anti-aging benefits is not feasible for most people over long periods of time and could lead to undesirable side effects (Longo et al., 2015).

Dietary fasting has also been studied for its impact on health and aging. There are many forms of fasting but common types include intermittent fasting (IF), which involves cycling between ad libitum feeding and periods of fasting, and time restricted fasting (TRF), in which food intake is limited to 6–12 h each day without a change in the total calorie intake of the normal diet (Hwangbo et al., 2020). Model studies has shown IF to lead to improved health with some reports of improved lifespan as well (Goodrick et al., 1990; Honjoh et al., 2009; Catterson et al., 2018; de Cabo and Mattson, 2019). TRF has also shown the potential to improve health and extend lifespan (Hwangbo et al., 2020). Compared to CR, the mechanisms relating fasting to aging are not as well-understood and results from rodent studies signify that IF and TRF effects on aging act through differing pathways. There have been proposed models, however, linking CR and IF through common key metabolic pathways (such as mTOR, IIS, and sirtuin) (Pan and Finkel, 2017). One relatively well-understood mechanism is that in the yeast Saccharomyces cerevisiae, in which fasting causes the activation of the stress resistance transcription factors Msn2/4 and Gis1 that regulate many protective and metabolic genes (Wei et al., 2008). In general, though, fasting is believed to be a time in which the organism activates alternative metabolic pathways which are important for repair and health maintenance (fasting physiology) (Longo and Panda, 2016). IF and TRF allow the organism to spend more time in this metabolic state which could contribute to greater health and lifespan. Human studies have found IF and TRF to be safe and to have the ability to improve metabolic health and physiological function, especially in obese individuals (Hwangbo et al., 2020; Martens et al., 2020). Yet, there are still dangers accompanying fasting implementation in certain populations of patients, especially those low in BMI, frail and old, and patients with diabetes receiving insulin or insulin-like drugs.

Although restrictions in total protein is associated with increased health and lifespan, studies have found that restrictions in specific amino acids (such as methionine or branched chain amino acids) cause similar effects. The benefits observed with methionine restriction in mice are also like those seen with CR (Brown-Borg, 2016). The longevity benefit of methionine restriction relies on GH signaling, as in the absence of GH signaling no lifespan extension is observed (Brown-Borg et al., 2014). Dietary amino acid levels are sensed by at least two evolutionarily conserved mechanisms—mTOR and GCN2 (general control non-derepressible two). The GCN2 pathway becomes activated by the absence of many amino acids while mTOR is activated by specific dietary amino acids (Li W. et al., 2014). mTORC1, a serine/threonine kinase subunit of mTOR, has been heavily studied and is an important regulator of cell growth and metabolism based on nutrient and growth factor levels (Kim and Guan, 2019). Pharmacologic inhibition of mTORC1 extends lifespan in mice and reduced levels of methionine leads to similar decreased activity in mTORC1 (Harrison et al., 2009). The conversion of methionine to S-adenosylmethionine (SAM), and the downstream SAM pathway, has been identified as the link between methionine and mTORC1 signaling. In short, less dietary intake in methionine leads to decreased mTORC1 signaling and increased health and lifespan (Kitada et al., 2019). Importantly, mice on LPHC diets in the Geometric Framework study showed low circulating branched chain amino acids which correlated with decreased activation of hepatic mTOR (Solon-Biet et al., 2014). This highlights mTOR as an important pathway in regulating health and lifespan.

Neurovascular coupling (NVC) disruptions and cerebral microvasculature changes are also being studied as potential targets for pharmacological therapies of the cognitive decline typically seen with aging, but dietary intervention offers another avenue of research into this topic. Increases in ROS production (especially mitochondrial-derived) as well as decreased production of oxidative stress protecting molecules, such as antioxidants and anti-inflammatories, are associated with vascular aging and disease (Ungvari et al., 2011, 2018, 2019; Springo et al., 2015). Contrarily, elevated levels of certain antioxidant enzymes, such as mitochondrial catalase, are associated with protection of cerebral microvasculature and the NVC mechanism in aging mice (Csiszar et al., 2019). As previously discussed, integration of dietary changes like caloric restriction and fasting increase an organism's stress response pathways which are known for their anti-inflammatory and health maintenance qualities. This raises an interesting question as to what role diet could have on maintenance and/or improvement of NVC and cerebral microvasculature in older adults and AD patients.

## Conclusion

Aging is a major risk factor for the development of neurodegenerative diseases such as Alzheimer's Disease (AD). Current therapeutic approaches toward the treatment of AD have proved to be ineffective and emphasize the need for alternative options. Certain medications (such as Rapamycin and Metformin) are intriguing because of their known safety in humans as well as their ability to improve health and lifespan in model organisms, but dietary interventions may be the most feasible and yet understudied option. Diet has long been known to impact human health and following certain diets (such as Mediterranean) is associated with decreased incidence of neurodegenerative diseases as well as increased health and lifespan. Early findings from a few small studies show that dietary changes (such as ketogenic diet) may be useful in the management of AD. However, much more work is needed to examine the plausibility of dietary interventions for the management of certain neurodegenerative diseases.

## Author Contributions

MT and HB-B wrote and edited the manuscript. Both authors contributed to the article and approved the submitted version.

## Conflict of Interest

The authors declare that the research was conducted in the absence of any commercial or financial relationships that could be construed as a potential conflict of interest.
